# Immunization of mice with YscF provides protection from *Yersinia pestis *infections

**DOI:** 10.1186/1471-2180-5-38

**Published:** 2005-06-24

**Authors:** Jyl S Matson, Kelly A Durick, David S Bradley, Matthew L Nilles

**Affiliations:** 1 Department of Microbiology and Immunology, University of North Dakota School of Medicine and Health Sciences, Grand Forks, ND 58202, USA

## Abstract

**Background:**

*Yersinia pestis*, the causative agent of plague, is a pathogen with a tremendous ability to cause harm and panic in populations. Due to the severity of plague and its potential for use as a bioweapon, better preventatives and therapeutics for plague are desirable. Subunit vaccines directed against the F1 capsular antigen and the V antigen (also known as LcrV) of *Y. pestis *are under development. However, these new vaccine formulations have some possible limitations. The F1 antigen is not required for full virulence of *Y. pestis *and LcrV has a demonstrated immunosuppressive effect. These limitations could damper the ability of F1/LcrV based vaccines to protect against F1-minus *Y. pestis *strains and could lead to a high rate of undesired side effects in vaccinated populations. For these reasons, the use of other antigens in a plague vaccine formulation may be advantageous.

**Results:**

Desired features in vaccine candidates would be antigens that are conserved, essential for virulence and accessible to circulating antibody. Several of the proteins required for the construction or function of the type III secretion system (TTSS) complex could be ideal contenders to meet the desired features of a vaccine candidate. Accordingly, the TTSS needle complex protein, YscF, was selected to investigate its potential as a protective antigen. In this study we describe the overexpression, purification and use of YscF as a protective antigen. YscF immunization triggers a robust antibody response to YscF and that antibody response is able to afford significant protection to immunized mice following challenge with *Y. pestis*. Additionally, evidence is presented that suggests antibody to YscF is likely not protective by blocking the activity of the TTSS.

**Conclusion:**

In this study we investigated YscF, a surface-expressed protein of the *Yersinia pestis *type III secretion complex, as a protective antigen against experimental plague infection. Immunization of mice with YscF resulted in a high anti-YscF titer and provided protection against i.v. challenge with *Y. pestis*. This is the first report to our knowledge utilizing a conserved protein from the type III secretion complex of a gram-negative pathogen as a candidate for vaccine development.

## Background

*Yersinia pestis*, the causative agent of plague, causes rapidly progressing disease in humans with a high mortality rate, especially in the pneumonic form of the disease. Due to the severe nature of plague, its ability for aerosol transmission, and the potential for human to human transmission plague is considered to be a disease of high concern as an agent of biological warfare or biological terrorism [1]. For this reason, an improved vaccine for plague is desirable. Current efforts for vaccine development have focused on two proteins: LcrV (also known as the V antigen) and the capsular F1 antigen [2]. The best results to date have been obtained by using a combination of recombinant LcrV and F1 subunits [3] separately or as a fusion protein [4,5]. These subunit vaccines demonstrate very good protection against both pneumonic and systemic forms of plague [2] in mouse models. One of the potential limitations of these subunit vaccines is that F1 is not required for full virulence of *Y. pestis*, as F1-negative strains have the same LD_50 _value as F1-positive strains [6-9]. A second limitation that could result in undesired side-effects in immunized individuals is the demonstrated immunosuppressive effect of LcrV [10-13]. Additionally, serologic diversity of LcrV has been reported, in *Yersinia *species other than *Y. pestis*, that could theoretically limit the usefulness of an LcrV based vaccine. While the recombinant subunit vaccines are very effective in experimental animals and offer protection against F1 minus strains of *Y. pestis *[2], the inclusion of other antigens with the LcrV/F1 subunit vaccine candidates could improve the ability of the resulting vaccine to offer protection against multiple *Y. pestis *strains, or the new antigens could be developed as separate vaccine candidates.

The type III secretion apparatus encoded on the low-calcium response (LCR) virulence plasmid, pCD1 in strain KIM [14], of *Y. pestis *is a conserved virulence mechanism that is absolutely required for virulence of *Y. pestis *[15]. YscF is a surface localized protein that is required both to secrete Yops and to translocate toxins into eukaryotic cells [16-19]. One report speculates that YscF polymerization is required for a YscF needle to puncture eukaryotic cell membranes [18]. Other researchers suggest that YscF and its homologs function to provide a base that a translocon complex is built upon, or that YscF builds a conduit from the bacterium to the eukaryotic membrane [20]. This suggestion seems more likely given that other proteins such as YopB, YopD, and LcrV are also required for translocation into eukaryotic cells [21-28]. Additionally, YscF needle producing *Y. enterocolitica *deficient in production of the translocators (LcrV, YopB, and YopD) do not translocate Yops into macrophages, demonstrating that the YscF-needle is not sufficient for translocation [19].

Most currently described pathogenesis-related type III secretion systems possess homologs to YscF. In pathogenic *Salmonella *and *Shigella*, the YscF homologs (PrgI and MxiH, respectively) have been demonstrated to form a needle structure that protrudes from the surface of bacterial cells [29-31]. The best-characterized homolog of YscF is EscF of enteropathogenic *E. coli *(EPEC). EscF is required for "attaching and effacing" (A/E) lesion formation on the intestinal mucosa and for type III secretion of effector proteins [32-34]. EscF is thought to be a structural component of the needle complex on the bacterial surface as it binds EspA, the major component of a filamentous surface organelle, and is required for formation of the EspA filaments. [32-34] However, this surface localization has never been visualized directly, as the only EscF antiserum generated was unable to recognize the native protein [33].

Based on the fact that YscF is thought to be a surface-expressed protein in *Y. pestis *and is required for virulence, we sought to determine if YscF could serve as a protective antigen against experimental plague infection. Immunization of mice with His-tagged YscF resulted in a high anti-YscF titer and significant protection against i.v. challenge with *Y. pestis*. The findings of this study suggest that YscF may be a potential plague vaccine candidate that could be used in conjunction with other plague antigens, or possibly alone if its efficacy can be improved by alternative delivery methods.

## Results and discussion

### Expression and purification of HT-YscF

**Figure 1:**
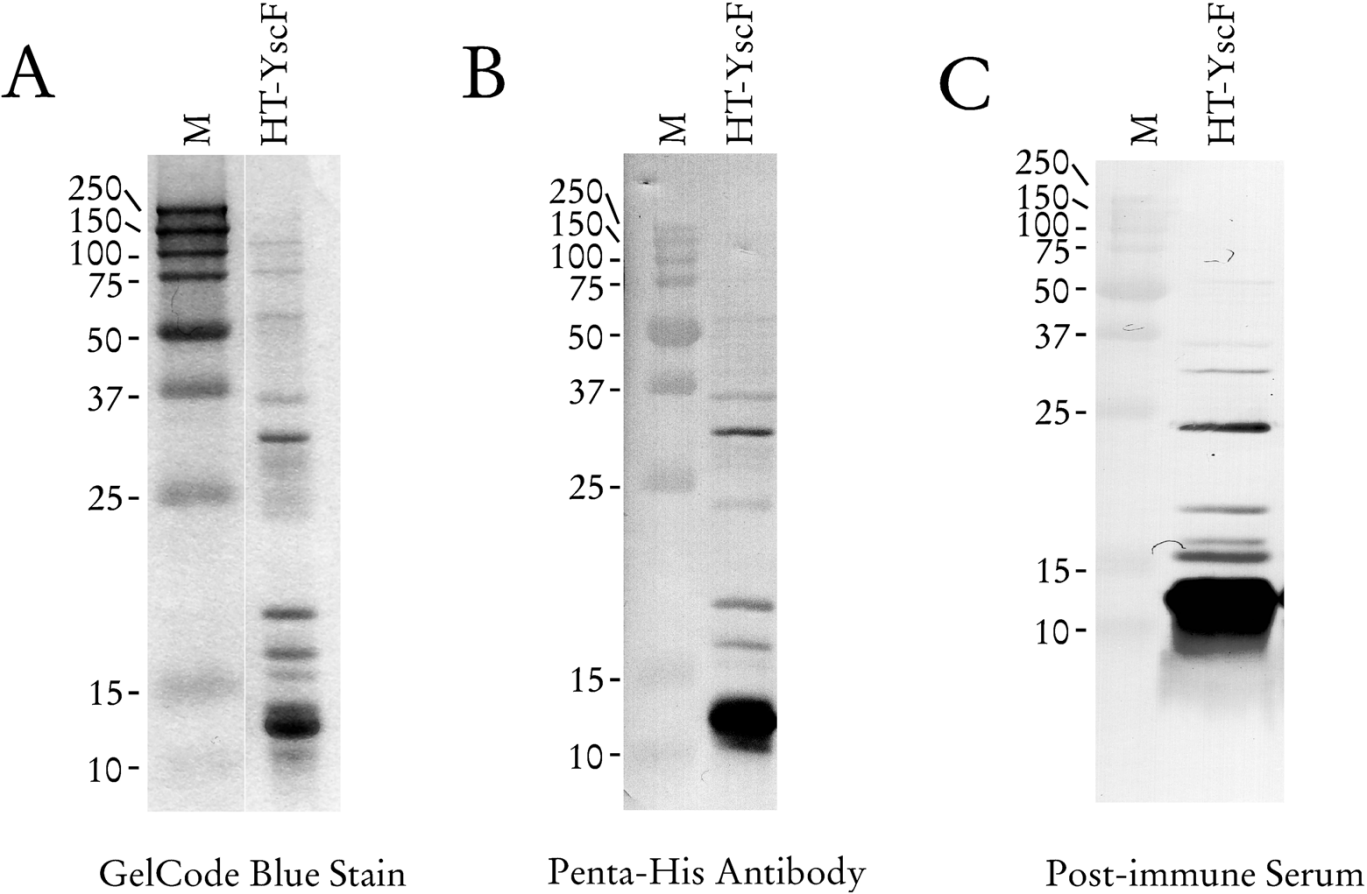
Purified HT-YscF was run on SDS-PAGE gels (12.5% for Panels A and B, 15% Panel C) to analyze purity of the recovered protein. Panel A, separated proteins were stained with GelCode Blue stain (Pierce Chemical). Panel B, purified HT-YscF was immunoblotted to PVDF and probed with antibody specific for penta-histidine to identify which bands contained the His-tag. Panel C, purified HT-YscF was immunoblotted to PVDF and probed with post-immune serum from immunized mice.

To facilitate the purification of YscF, *yscF *was cloned into the overexpression plasmid pET24b (Novagen) to yield a hexahistidine-tag on the C-terminus of YscF (HT-YscF). *E. coli *BL21(DE3) harboring the HT-YscF expression plasmid, pJM119, was grown in one liter of LB broth [35] containing kanamycin at 37°C. Expression of HT-YscF was induced after 2 h of growth with 0.3 mM IPTG and then incubated until the A_550 _reached ~1.0. Cells were harvested by centrifugation and disintegrated by passage through a French pressure cell at 20,000 lb/in^2^. Subsequent to disintegration, the extracts were clarified by centrifugation at 3,200 × *g *for 20 min at 4°C. Affinity purification of HT-YscF was performed using Talon resin (BD Clontech) using standard methods. Purity of the recovered protein (> 95 %) was estimated by SDS-PAGE on a 15% (wt/vol) gel followed by staining with a Coomassie blue stain. The purified HT-YscF ran as multiple bands on the gel, regardless of the presence or absence of a reducing agent, such as dithiothreitol (data not shown). A band that corresponded to the predicted size of HT-YscF was the dominant species and other larger bands could also be visualized (Figure 1A). Based on the sizes of these bands, it is likely that they represent dimers and other multimers of YscF. The presence of YscF multimers is not surprising as YscF and its homologs are known to form multimeric structures [29,36]. Support for the contention that the larger bands are multimers of YscF is seen in Fig. 1B. Purified recombinant HT-YscF was immunoblotted with a Penta-His specific antibody (Qiagen). The results seen in Figure 1 show that the larger bands seen in panel A are also recognized by the penta-His antibody (Fig. 1B) demonstrating that the larger bands are likely recombinant proteins containing a poly-histidine tag and, therefore, contain YscF. Additionally, the purified HT-YscF was immunoblotted and probed with serum from immunized mice (Fig. 1C) to demonstrate that the immunized mice mounted an immune response against the purified HT-YscF. The larger bands seen in the Coomassie stain and visualized on the immunoblots (with both anti-Penta-His antibody and post-immune sera) could also be contaminating *E. coli *proteins, that cross-react to the penta-His antibody, this possibility cannot be currently discounted. The presence of the higher molecular weight proteins, if they are not YscF multilmers and they are from *E. coli*, likely had little influence on this study as immunization with HT-YscF induced a specific antibody response to native YscF produced by *Y. pestis *(discussed below).

### Specificity of the antibody response to YscF

**Figure 2:**
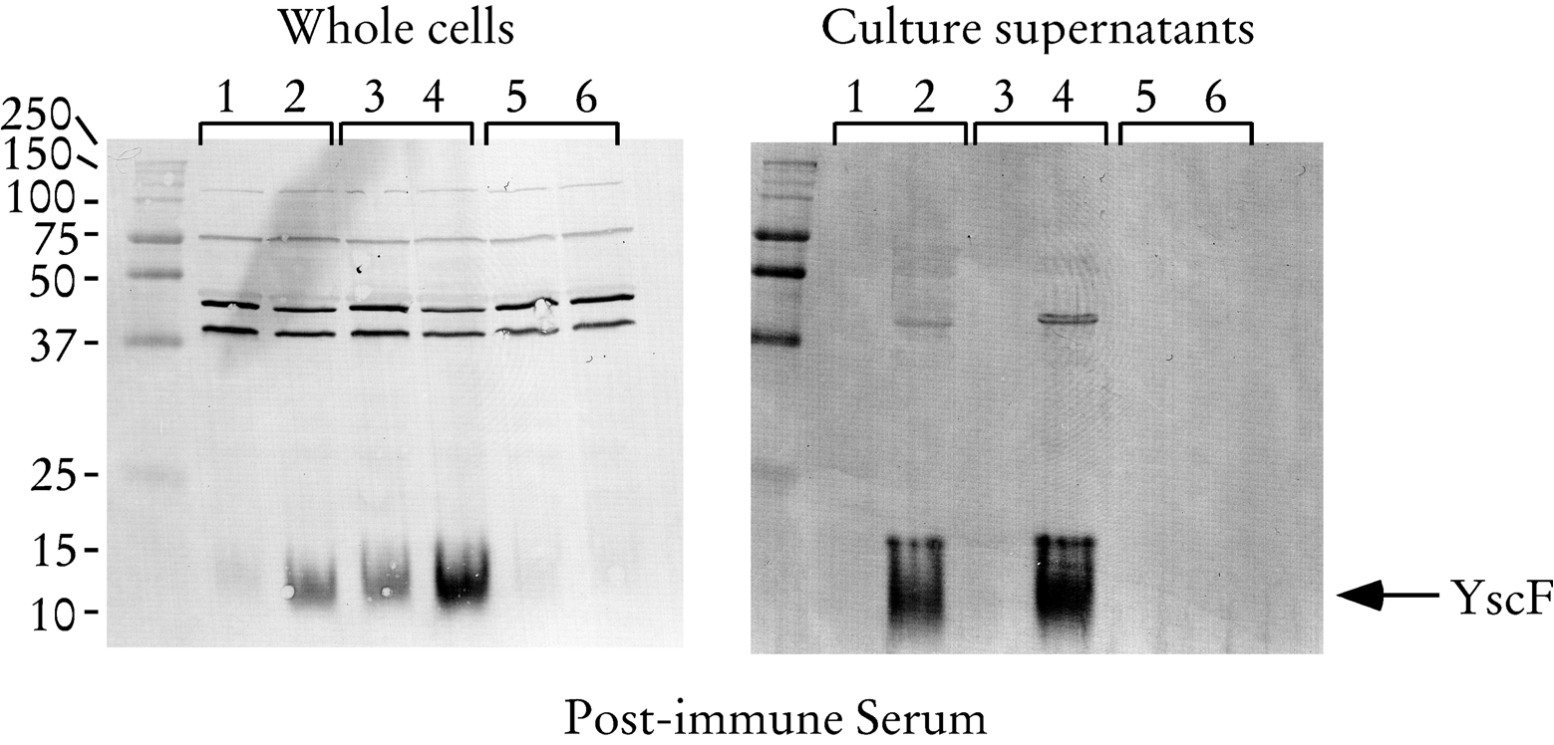
Derivatives of *Yersinia pestis *KIM8-3002 (KIM5 pPCP1-minus, Sm^r^) were grown in a chemically defined medium at 26°C for 2 h in the presence (lanes 1, 3, and 5) or absence of calcium (lanes 2, 4, and 6). Lanes 1 and 2 contain *Y. pestis *KIM8-3002. Lanes 3 and 4 contain *Y. pestis *KIM8-3002 Δ*yscF *expressing YscF from pBAD18-YscF. Lanes 5 and 6 contain *Y. pestis *KIM8-3002 Δ*yscF *gene. After the 2 h growth, the culture was shifted to 37°C to induce expression of the Ysc type III secretion system and the Low Calcium Response. Following 4 h of growth at 37°C cultures, were centrifuged to obtain whole cell fractions and cell-free culture supernatant fractions. Total proteins from each fraction were precipitated with 10% trichloro acetic acid. Dried proteins were resuspended in SDS-PAGE sample buffer and electrophoresed in a 15% SDS-PAGE gel. Proteins were then transferred to a PVDF membrane and immuno-blotted with pooled mouse serum used at a 1:20,000 dilution. Mouse serum was obtained by bleeding mice subsequent to immunization with HT-YscF, serum from several mice was pooled to control for animal specific variation. The position and sizes for the molecular weight markers are indicated and the position of YscF is shown.

To confirm that the recombinant HT-YscF antibody response generated in immunized mice was directed against YscF from *Y. pestis*, *Y. pestis *protein extracts from the parental strain KIM8.3002 (pgm^-^, pla^-^) and extracts from a isogenic *yscF *deletion strain (kindly provided by G. Plano, University of Miami, Miami, Fl.) were immunoblotted and probed with pooled antiserum from HT-YscF immunized mice (Figure 2). The results in Figure 2 demonstrate that the pooled mouse antiserum specifically recognizes YscF produced by *Y. pestis*. As seen in Figure 2, YscF is visualized on the immunoblot as a highly reactive band of the correct predicted size and the YscF band is only seen only in strains containing the *yscF *gene (Figue 2, lanes 1–4). Importantly, no bands are seen in lanes 5 and 6 that contain proteins derived from the *yscF *deletion strain. In lanes 1 and 2, calcium regulation of the YscF band is seen as expected for the LCR-regulated *yscF *gene. Transcomplementaion of the Δ*yscF *strain with pBAD-YscF (G. Plano, University of Miami) restored YscF reactivity to the HT-YscF serum in the Δ*yscF *strain (Figure 2, lanes 3 and 4). Demonstrating that the lack of YscF reactivity in the Δ*yscF *strain was due to the deletion of *yscF*. The higher molecular weight bands seen in the whole cell fraction (Figure 2, lanes 1–6) represent cross-reactive *Y. pestis *bands not specific to YscF, as they are present in the Δ*yscF *samples. The same cross-reactive bands are also present in whole cell samples probed with pre-immune serum (data not shown) suggesting that the reactivity seen is not induced by immunization with HT-YscF. The higher molecular weight band seen in the culture supernatant fractions could represent a multimer of YscF (possibly a trimer) or YscF in complex with another secreted protein. This higher molecular weight species is likely related to YscF as a similar band is not seen with pre-immune sera (data not shown) or in the Δ*yscF *strain (Figure 2). These results demonstrate that mice immunized with recombinant HT-YscF produce antibodies that specifically recognize YscF produced by *Y. pestis*.

### Active immunization of outbred mice followed by challenge with *Y. pestis *KIM5

To examine the ability of HT-YscF to protect mice from *Y. pestis *infection outbred mice were immunized intra peritoneally with purified HT-YscF using complete Freund's adjuvant (CFA) for primary immunization and incomplete Freund's adjuvant (IFA) for booster immunizations or with a phosphate-buffered saline (PBS, [37]) control in CFA or IFA to control for adjuvant effects. For these studies, 6-to 8-week-old female Swiss-Webster mice were immunized i.p. with 40 μg/mouse HT-YscF in PBS or PBS (control mice) alone emulsified 1:1 with CFA. Experimental mice were boosted with 40 μg/mouse HT-YscF in IFA after two weeks and with 20 μg/mouse HT-YscF in IFA at 4 weeks post-immunization. Negative control mice were boosted with PBS emulsified with IFA according to the same schedule. Two weeks following the final booster immunization, sera were collected from the HT-YscF-immunized and the PBS-immunized mice to assay for HT-YscF reactivity. Sera from 22 mice from the HT-YscF-immunized and the PBS-immunized groups were tested for total IgG reactivity. HT-YscF immunized mice had a geometric mean titer of 1:40,000 for IgG specific to HT-YscF while PBS-immunized mice had HT-YscF titers less than the lowest dilution 1:800 (Table 1). After establishing that the HT-YscF immunized mice had developed a strong antibody response to HT-YscF, the mice were challenged with *Y. pestis*. Two weeks after the final immunization, groups of 10 mice were challenged intra venously via the retro-orbital sinus with 10^1 ^to 10^6 ^CFU *Y. pestis *KIM5 (pgm^-^) in PBS (grown at 26°C). The mice were observed for 19 days after challenge, and the average doses required to kill 50% of the mice (LD_50_) for the treatment groups were calculated using the extrapolation method of Reed and Muench [38]. Mice that were immunized with HT-YscF demonstrated a 134-fold increase in the calculated LD_50 _value as compared to PBS-immunized mice (Table 1). These results demonstrate that immunization of mice with HT-YscF was able to provide a degree of protection to the immunized mice from a subsequent challenge with *Y. pestis *(Table 1). While the protection provided by HT-YscF is not of the same magnitude as that seen with the protective antigens F1 or LcrV, the increased LD_50 _value clearly shows that immunization with YscF affords a significant level of protection. Thus, HT-YscF becomes the only other reported antigen apart from LcrV or F1 to induce a significant protection in F1-positive *Y. pestis*. Immunization with YopD has been demonstrated to provide significant protection against a F1-minus mutant of *Y. pestis *[39]. The protection generated by HT-YscF suggests that YscF could be potentially developed as a novel subunit vaccine for *Y. pestis *or could serve as an additional antigen in a multivalent *Y. pestis *vaccine comprised of YscF, F1, and LcrV for example.

### Characterization of the antibody response to HT-YscF

Mice immunized with HT-YscF demonstrated a strong antibody response to YscF and provided protection to the vaccinated mice from lethal *Y. pestis *challenge. Due to the strong antibody response, isotyping analysis was performed to determine the predominant isotypes of antibodies produced by mice in response to vaccination with HT-YscF in CFA/IFA. Anti-YscF antibody titers were determined two weeks following the last immunization, prior to challenge. The YscF-specific antibody titers of PBS-immunized mice were below the ELISA assay baseline of 1:800 (Table 1), as was the pre-immune serum (data not shown). However, the HT-YscF immunized mice reached a GMT (geometric mean titer) of 1:40,000 (Table 1) for total IgG. Isotyping analysis was performed on the pooled sera obtained from the 22 mice selected for total the IgG analysis reported in Table 1. Pooled sera were used to minimize the animal to animal variation expected from using outbred mice. The isotyping analysis demonstrated no significant IgA or IgM production (Table 2) and a very high IgG titer (Table 2) as expected from the data in Table 1. Among the IgG subclasses IgG2b appeared to have the highest levels (Table 2), although IgG1 and IgG2a levels were also very high (Table 2). IgG3 levels were the lowest (Table 2). Generally, immunization with CFA tends to drive a strong Th1 response. Immunization with HT-YscF in CFA induced a strong Th1 response, evident by the high IgG2a response. However, a strong Th2 response is also present as seen by the high IgG1 and IgG2b levels. Immunization with HT-YscF induced a strong IgG1 response in mice and interestingly, Titball et al. have shown that high IgG1 titers to a F1/LcrV recombinant subunit vaccine correlated very well with protection against pneumonic plague in mice [40]. This suggests that YscF, which also induces a strong IgG1 response, could possibly afford some protection against pneumonic challenge as well as against systemic challenge.

### Ability of α-YscF to effect Yop translocation

To examine one possible method that anti-YscF could be functioning to provide protection in immunized animals the ability to translocate Yops in the presence of anti-YscF antiserum was examined. Antibody to the surface-localized LcrV has been shown to block the ability of the TTSS in *Y. pestis *to translocate Yops into cultured macrophages [41,42] but anti-LcrV was unable to block translocation into HeLa cells [21].

However, anti-LcrV was able to block Yops translocation by *Y. pseduotuberculosis *into HeLa cells [26]. Since YscF is also surface-localized the ability of anti-HT-YscF to block Yop translocation into HeLa cells was tested. Day et al have described an elegant methodology to follow the translocation of Yop effector by fusing them to a Elk reporter [43]. Elk, a eukaryotic transcriptional activator, becomes phosphorylated only after entering the nucleus, providing a reporter for translocation into eukaryotic cells [43,44]. This methodology has the advantage of not requiring cell fractionation and protease protection assays to establish the intracellular localization of translocated proteins. To test the ability of anti-YscF to block translocation *Y. pestis *KIM8-3002 was transformed with plasmid pYopE_129_-Elk [43]. HeLa cells were infected at an MOI of 10 and infection was allowed to progress for 4 h. After the 4 h incubation infected HeLa cells were harvested and immunoblotted to analyze YopE, Elk and PO_4_-Elk production. *Y. pestis *KIM8-3002 (wt; [24]) and an isogenic translocation defective strain containing a *yopD *deletion (KIM8-3002.2, Δ*yopD*; [45]) both containing pYopE_129_-Elk were used as positive and negative translocation controls, respectively. Immunoblots of HeLa cells infected with the wt and the Δ*yopD *strains showed that only the wt strain elicited the production of PO_4_-Elk while the Δ*yopD *strain had no production of PO_4_-Elk. The wt and the Δ*yopD *strains were used to infect HeLa cells in the presence of a 1:10 or a 1:25 dilution of anti-YscF (titer for HT-YscF, 1:100,000) or in the presence of anti-PcrG (a *Yersinia *non-reactive antibody control, titer for PcrG, 1:20,000). The wt strain was capable of translocating YopE-Elk in presence of both anti-sera, demonstrating that anti-YscF was not capable of blocking Yops translocation and expectedly the Δ*yopD *strain was still defective for translocation. The experiment likely contained sufficient antibody against YscF to block translocation. In a previous report Pettersson et al used as low as a 1:100 dilution of an anti-LcrV anti-sera with a titer of 1:20,000 for LcrV and in that experiment translocation of Yops into HeLa cells was blocked [26]. These results suggest that antibody to YscF may not exert its protective effect by blocking Yops translocation. The results also suggest that while YscF is surface-exposed in the yersinae, antibody directed against YscF, unlike, anti-LcrV cannot block translocation. This may imply that YscF activity is shielded from neutralization by antibody, unlike LcrV activity that is blocked by antibody in some cases. However, since anti-LcrV was unable to block Yops translocation into by *Y. pestis *into HeLa cells [21] but could block translocation into cultured macrophages [41,42], the possibility remains that anti-YscF also display this type of differential blockage.

## Conclusion

**Figure 3:**
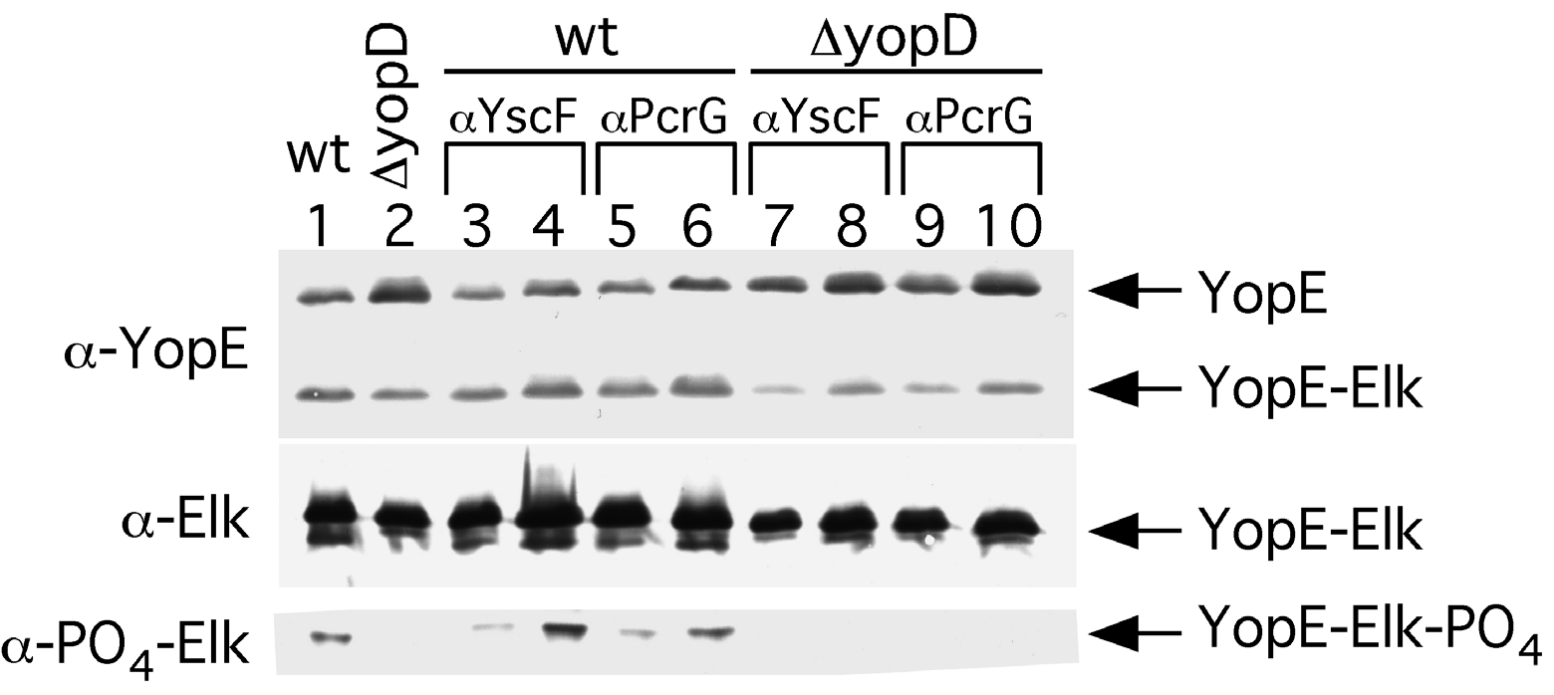
*Y. pestis *strain KIM8-3002 (wt) (lane 1 and lanes 3–6) and KIM8-3002.2 (Δ*yopD*) (lane 2 and lanes 7–10) both containing plasmid pYopE_129_-Elk were used to infect HeLa cells at an MOI of 10. 4 h following infection the culture supernatant containing non-adherent bacteria were removed the remaining adherent HeLa cells were solubilized in 2X SDS-PAGE buffer. Following solubilization proteins were separated and immunoblotted to triplicate PVDF membranes. The triplicate blots were probed with α-YopE, α-Elk, or α-PO_4_-Elk, followed by incubation with an alkaline phosphatase conjugated secondary antibody and developed using NBT/BCIP. To some samples anti-sera specific for HT-YscF (lanes 3–4 and lanes 7–8) or the *Pseudomonas *protein, PcrG (lanes 5–6 and lanes 9–10), were added at dilutions of 1:10 (lanes 3, 5, 7, and 9) or 1:25 (lanes 4, 6, 8, and 10).

In this study we have determined that immunization of mice with recombinant YscF can protect mice from an i.v. challenge with *Y. pestis*. This is the first report to our knowledge that has utilized a conserved protein from the type III secretion complex of a gram-negative pathogen as a candidate for vaccine development. This result suggests that type III secretion complexes of other gram negative pathogens could also serve as vaccine targets. YscF and its homologs are obvious targets for use as vaccine candidates as they are surface exposed and are required for virulence in all the systems examined. The protective antibody response elicited by HT-YscF is evidence that YscF is not only expressed during the course of a plague infection, but is also in a location accessible to antibodies at some point in the infectious process. The mechanism of protection by the YscF antibody response is currently under investigation. Essentially three possibilities exist to explain the antibody's protective activity: increased opsonization of the bacteria, enhanced complement binding to the bacterial surface, or direct blocking of Yops translocation into the host cells. Cytotoxicity assays (data not shown) and the ability to translocate Elk-tagged YopE into HeLa cells (Figure 3) have shown that it is unlikely that anti-YscF directly blocks Yops translocation into HeLa cells. Suggesting that a blockage of Yops translocation may not be the mechanism whereby anti-YscF antibodies are protective. The degree of protection observed after immunization with YscF is not as great as that seen for the two known protective antigens, F1 and LcrV. This result suggests that YscF could be best used in combination with the other known antigens to formulate a tri-valent vaccine for *Y. pestis*. However, further work could lead to the development of YscF as a monovalent vaccine or combined with other antigens that could be efficacious not only against *Y. pestis*, but also against *Y. enterocolitica *and *Y. pseudotuberculosis*.

## Methods

### Cloning of *yscF *for overexpression and HT-YscF purification

Plasmid pJM119 was constructed by cloning a *Bam*HI-and *Xho*I-cleaved PCR product into pET24b (Novagen, Madison, WI). The primers used to amplify *yscF *were HT-YscF Start (5' CGG GAT CCG ATG AGT AAC TTC TCT GGA TTT 3') and HT-YscF Stop (5' CCG CTC GAG TGG GAA CTT CTG TAG GAT GCC 3'). *E. coli *BL21(DE3) (Novagen) harboring pJM119 was used for HT-YscF overpexpression according to the manufacturer's suggestions. HT-YscF was purified using Talon resin (BD Clontech, Palo Alto, CA) according the manufacturer's directions.

### Immunization of mice and infection with *Y. pestis *KIM5

For primary immunization 6-to 8-week-old female Swiss-Webster mice were immunized i.p. with 40 μg/mouse His-tagged YscF or phosphate-buffered saline [37] PBS (control mice) emulsified 1:1 with complete Freund's adjuvant (CFA). Booster immunizations were performed the same as the primary immunization with the substitution of Incomplete Freund's Adjuvant for CFA. Mice were challenged with *Y. pestis *via the retro-orbital sinus using blunt-feeding needles. *Y. pestis *used to infect mice was grown overnight in HIB broth, sub-cultured to an A_620 _of 0.2 absorbance units and grown with shaking to an A_620 _of 1.0 absorbance unit. *Y. pestis *cells for infection were harvested by centrifugation and resuspended in PBS. Plate counts were performed to verify CFUs for the infectious doses. Infected animals were monitored for death for up to 19 days, after which survivors were euthanized by CO_2 _inhalation, according to the guidelines of the Panel on Euthanasia of the American Veterinary Medical Association. All animal work for this project was reviewed and approved by UND's IACUC.

### Bacterial strains, growth and fractionation

Bacterial strains used were KIM8-3002 [24], Δ*yscF *an isogenic in-frame deletion of *yscF *(G. Plano, University of Miami), and KIM8.3002.2 Δ*yopD *[45]. *Y. pestis *strains were grown in heart infusion broth or on tryptose blood agar base medium (Difco Laboratories, Detroit, MI) at 26°C for genetic manipulations. For physiological studies, growth of *Y. pestis *was conducted in a defined medium, TMH, as previously described [46]. Bacterial cells were fractionated as previously described [24]. Briefly, bacterial cells were chilled on ice after growth, harvested by centrifugation, and washed in cold phosphate-buffered saline (PBS; [37]). Bacterial whole cell fractions were prepared by resuspending the washed cells in cold PBS and precipitating total proteins with 10% (vol/vol) trichloroacetic acid (TCA) on ice overnight. Secreted proteins were recovered from the bacterial growth medium by centrifuging the spent medium a second time, transferring the supernatant to a clean tube, and precipitating with 10% (vol/vol) TCA on ice overnight. The TCA-precipitated proteins were pelleted by centrifugation (20,800 × g at 4°C) for 20 min and resuspended in 2X sodium dodecyl sulfate (SDS) sample buffer [37].

### Protein electrophoresis, visualization and immunodetection

Proteins were separated by SDS-polyacrylamide gel electrophoresis (SDS-PAGE), using 12.5 % or 15 % (wt/vol) polyacrylamide gels according to the method of Laemmli [47]. Samples were boiled 3–5 min before loading on the gels. Samples were loaded such that lanes containing different culture fractions represented equivalent amounts of the original cultures. Proteins were visualized in gels using GelCode Blue stain (Pierce Chemical, Rockford, IL) according to directions. For immunoblots, proteins resolved by SDS-PAGE were transferred to Immobilon-P membranes (Millipore Corp., Bedford, MA) using carbonate transfer buffer (pH 9.9) [48]. Specific proteins were visualized using mouse or rabbit polyclonal antibodies specific for YopE (rabbit α-YopE; gift from G. Plano, University of Miami, Miami, FL), YscF (mouse α-YscF, this study), Elk (rabbit α-Elk, Cell Signaling Technology, Beverley, MA) and PO_4_-Elk (rabbit α-PO_4_-Elk, Cell Signaling Technology). Hexahistidine tagged YscF was visualized using a penta-histidine specific antibody (mouse α-Penta-His, Qiagen, Valencia, CA) Alkaline phosphatase-conjugated secondary antibody (goat anti-rabbit immunoglobulin G or goat anti-mouse immunoglobulin G; Pierce) was used to visualize proteins by development with nitroblue tetrazolium and 5-bromo-4-chloro-3-indolylphosphate (NBT-BCIP; Fisher Scientific, Fair Lawn, NJ).

### Antibody characterization and isotyping

Flat-bottom, 96-well Nunc Maxisorp immunoplates (Fisher Scientific, Pittsburgh, PA) were coated with 100 μl of HT-YscF solution (4 μg/ml in Binding solution (0.1 M NaH_2_PO_4_, ph 9.0) at room temperature for 2 h (or overnight at 4°C). The wells were blocked with 200 μl/well blocking buffer (1% bovine serum albumin in TTBS (tris-buffered saline [37] + 0.5% Tween 20) and washed with TTBS. Test sera were serially diluted in blocking buffer and 100 μl of each dilution was added to duplicate wells that were incubated for 2 h at RT (or overnight at 4°C). The plates were washed and incubated for 2 h at RT with alkaline-phosphatase-conjugated anti-mouse secondary antibody. For quantitation of YscF-specific immunoglobulin isotypes and subclasses the plates were coated with alkaline-phosphatase-labeled anti-mouse isotype-specific antibody (1:400 in blocking buffer; Southern Biotech, Birmingham, AL). The wells were washed and 75 μl 3 mM para-nitro phenyl phosphate (p-NPP) was added to each well. The plates were incubated for 15 min at RT and the reaction was stopped by the addition of 50 μl of 1.5 M NaOH to each well. A_405 _was measured to monitor the cleavage of p-NPP. Antibody titers were determined as reciprocal numbers of the highest serum dilution that displayed values for optical density twofold higher than the value of the control serum.

### Infection assays

Infection of eukaryotic cells was performed as described previously [24]. Prior to infection, eukaryotic cells were subcultured into 35-mm-diameter six-well tissue culture plates in RPMI-FBS and incubated at 37°C under 5% CO_2 _for 48 to 72 h to a density of 5 × 10^5 ^to 8 × 10^5 ^cells per well. Cells were washed twice with warm L15 lacking FBS immediately prior to infection. Bacteria were cultivated at 26°C in HIB and used at an OD_620 _of ~1.0 for tissue culture infections. Bacteria were added (at a multiplicity of infection (MOI) of 5 to 10) directly to prewarmed medium in the wells of the six-well plates. Plates were then centrifuged at 200 × g at RT for 5 min to achieve contact between the bacteria and the target cells and incubated at 37°C for 4 h.

### Translocation of YopE

Detection of Elk-tagged YopE from pYopE_129_-Elk was performed as described [43]. *Y. pestis *strains carrying plasmid pYopE_129_-Elk were used to infect HeLa cells. After 4 h, the culture supernatants were removed, and the infected adherent cells were lysed by the addition of 100 μl of 2X SDS-PAGE lysis buffer containing Pefabloc (Roche Molecular Biochemicals, Indianapoli, IN) and phosphatase inhibitor (P-2850) cocktail (Sigma, St. Louis, MO). Samples were boiled for 5 min and loaded onto 12.5 % SDS-PAGE gels, immunoblotted to PVDF membranes and probed with Elk-1 (#9182) or phosphospecific Elk-1 (#9181) antibody preparations (Cell Signaling Technology). Anti-sera specific for HT-YscF (titer of 1:100,000, this study) or the *Pseudomonas aeruginosa *LcrG homolog, PcrG (titer of 1:20,000, Matson and Nilles, unpublished), were used at dilutions of 1:10 or 1:25 in the infection medium to assess the ability of α-YscF to effect YopE translocation.

## Authors' contributions

J .S. M. cloned *yscF *for overexpression, purified HT-YscF, characterized the reactivity of antiserum to YscF, assisted with immunization and infection of mice, assisted with the LD_50 _calculation, and wrote the draft of the manuscript. K. A. D performed immunizations and infections of the mice and performed the antibody isotyping. D. S. B. helped to design the immunization protocol and edited the manuscript. M. L N. conceived of the study, supervised the work, calculated the LD_50 _and edited the manuscript.

## Figures and Tables

**Table 1:** IgG response to HT-YscF vaccination and LD_50 _determination.

Immunogen	anti-YscF GMT*	LD_50_	Fold increase in survival
PBS	< 1:400	159	-
HT-YscF	1:40,000	21,344	134

*Geometric mean titer for total IgG, determined from 22 HT-YscF immunized mice.

**Table 2:** Antibody isotype titers from mice* immunized with HT-YscF.

Antibody isotype	Titer
Total Ig	1:100,000
IgM	< 1:800
IgG1	1:100,000
IgG2a	1:100,000
IgG2b	> 1:100,000
IgG3	1:20,000
IgA	< 1:800

*Sera from 22 mice were pooled for this analysis.
